# Interface-mediated spontaneous symmetry breaking and mutual communication between drops containing chemically active particles

**DOI:** 10.1038/s41467-020-15713-y

**Published:** 2020-05-05

**Authors:** D. P. Singh, A. Domínguez, U. Choudhury, S. N. Kottapalli, M. N. Popescu, S. Dietrich, P. Fischer

**Affiliations:** 1 Max-Planck-Institut für Intelligente Systeme, Heisenbergstr. 3, D-70569 Stuttgart, Germany; 2 Department of Physics, Indian Institute of Technology Bhilai, Raipur, 492015 India; 3 Física Teórica, Universidad de Sevilla, Apdo. 1065, 41080 Sevilla, Spain; 4 IV. Institut für Theoretische Physik, Universität Stuttgart, Pfaffenwaldring 57, D-70569 Stuttgart, Germany; 5 Institut für Physikalische Chemie, Universität Stuttgart, Pfaffenwaldring 55, D-70569 Stuttgart, Germany

**Keywords:** Colloids, Fluids

## Abstract

Symmetry breaking and the emergence of self-organized patterns is the hallmark of complexity. Here, we demonstrate that a sessile drop, containing titania powder particles with negligible self-propulsion, exhibits a transition to collective motion leading to self-organized flow patterns. This phenomenology emerges through a novel mechanism involving the interplay between the chemical activity of the photocatalytic particles, which induces Marangoni stresses at the liquid–liquid interface, and the geometrical confinement provided by the drop. The response of the interface to the chemical activity of the particles is the source of a significantly amplified hydrodynamic flow within the drop, which moves the particles. Furthermore, in ensembles of such active drops long-ranged ordering of the flow patterns within the drops is observed. We show that the ordering is dictated by a chemical communication between drops, i.e., an alignment of the flow patterns is induced by the gradients of the chemicals emanating from the active particles, rather than by hydrodynamic interactions.

## Abstract (web summary)

Complex systems exhibit unique properties like spontaneous symmetry breaking and self-organization. Singh et al. show that catalytically active, non-propelling particles can induce steady vortical flows within a drop, as well as flow alignment between neighboring drops.

## Introduction

Active matter confined in drops can exhibit spontaneous symmetry breaking^1–3^ and can serve as a model for prebiotic protocells^4^. So far, the experimental realization of active drops has relied on building blocks with symmetry-breaking built in or which are highly motile. For instance, actomyosin solutions^2,5^, which also serve as a model for the cytoskeleton, contain motor proteins that show polar motion along actin fibers. Similarly, self-organization can arise when swimming bacteria, which again are highly motile and directional, are confined^6^.

Here, we consider chemically active particles (CAPs) that exhibit negligible motility by themselves. We show that even such simple building blocks can induce spontaneous symmetry breaking due to collective effects when confined within a drop. The mechanism is different from those which have been considered so far in active drops, in that it involves the response of the interface to the chemical activity of the particles. Even though the CAPs induce only small perturbations of the interfacial tension, these can nevertheless give rise to considerable hydrodynamic flows and relevant long-ranged effects. The ordering of flow patterns in an ensemble of such drops is captured by a theoretical model of long-ranged interdrop chemical communication. The combined distribution of chemicals produced by the active drops within the ensemble is seen to influence the flow alignment within each active drop, thus causing the communication between the drops.

## Results

### Chemically active particles

**Fig. 1:**
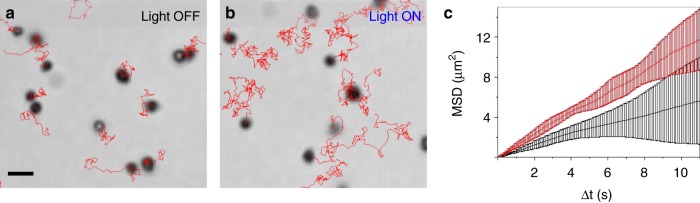
Low motility of the chemically active titania particles. Top-down view of the motion of TiO_2_ particles, sedimented at the bottom of a drop of hydrogen peroxide solution, at low areal number density (<4% of the area is covered by the particles): **a** standard Brownian motion in the absence of UV illumination; **b** enhanced diffusion due to self-propulsion upon illumination with UV light; **c** the mean squared displacement (MSD) determined from the particle trajectories as in **a** (black, lower part) and **b** (red, upper part) tracked over 15 s. The images in **a**, **b** show an area near the projection of the center of the drop onto the plane of the glass wall. The scale bar corresponds to 5 μm. The black disks correspond to the particles at the end of the tracked trajectories (red lines). In **c** the MSD(Δ*t*) for each of the tracked trajectory in the absence or presence of UV illumination, respectively, is located within the corresponding black or red shaded region. Therein, the red and black lines indicate the corresponding mean values.

The basic unit of our active-matter system is composed of irregularly shaped, photo-chemically active TiO_2_ particles with a linear extent of ca. 1 μm on average. The particles form a sedimented monolayer at the bottom of a sessile drop (of diameter ≈ 300 μm and height ≈ 100 μm) made of an aqueous solution of 3% (v/v) hydrogen peroxide (H_2_O_2_). The drop rests on a glass surface and is completely immersed within a thick film of silicone oil. (See the “Methods” section for details.) Upon sedimentation, the particles distribute uniformly in a monolayer over the bottom area of the drop (which is in contact with the glass wall). In the absence of illumination with UV light, the particles exhibit standard Brownian diffusion (see Fig. 1a) and the areal distribution of the particles remains uniform. However, when the system is exposed to UV light (wavelength 365 nm), photocatalytic decomposition of H_2_O_2_ occurs at the surface of the particles via UV light-induced formation of electron-hole pairs within TiO_2_, so that the particles become chemically active^7–11^.

### Dynamics within a single drop

Upon turning on the UV illumination the particles become chemically active. At low areal number densities of the particles one can resolve the two-dimensional directional motion of the particles, which can be ascribed to the particles having irregular, non-spherical shapes (compare Fig. 1a and b). This leads to an overall enhanced diffusion, as shown by the increase of the slope of the mean-squared displacement (see Fig. 1c). Due to the irregular shapes of the particles, as well as to a significant size dispersity, the velocity corresponding to self-propulsion varies significantly among the particles. The motion of each particle is seemingly independent of the presence of the others, as expected in the dilute limit (i.e., less than 4% of the area is covered by particles). The analysis of the results obtained from more than 50 trajectories of CAPs exhibiting directional motion provides a mean value for the self-propulsion velocity of ≈5 μm/s, with an upper limit of 8–10 μm/s.

**Fig. 2:**
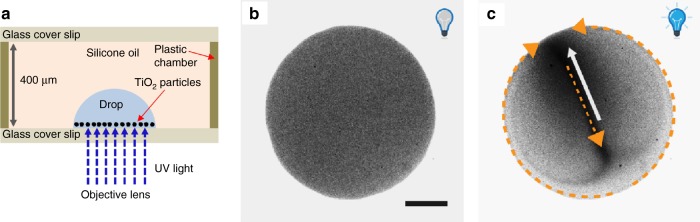
Emergence of self-organized vortical flow patterns in a drop. **a** Schematic representation (not to scale) of the experimental set-up. **b**, **c** Motion of TiO_2_ particles, forming a sedimented monolayer at the bottom of a drop of hydrogen peroxide solution, for a high areal number density (≈40% of the area is covered by the particles). Upon illumination with UV light, the equilibrium uniform distribution of the particles—at the bottom surface and in the absence of UV illumination (**b**)—evolves after a transient time to a steady flow pattern and collective motion of the CAPs as indicated schematically by the orange arrows (**c**). The scale bar corresponds to 100 μm. For the definition of the polarity **d** of the drop (white arrow in **c**), see the main text.

At sufficiently large values of the areal number density of the particles, the active Brownian dynamics of independent particles is replaced by the emergence of a steady, three-dimensional hydrodynamic flow pattern and an associated in-plane collective motion of the CAPs (see Fig. 2c). The spatially homogeneous distribution of the particles at the bottom surface is spontaneously broken and a characteristic distribution, with a flow pattern exhibiting two adjacent, counter-rotating vortices, sets in (see Fig. 2b, c and the Supplementary Movie 1): the particles are advected along the circumference of the drop base, so that the two streams merge into a single one. The latter is redirected along a diameter of the base of the drop up to the opposite side of the circumference, from where it splits into two streams along the circumference. The direction of the flows along the circumference is correlated with the spatial distribution of the areal number density of the particles along the symmetry diameter: the region of increased density is located near the point of confluence of the streams and noticeably displaced relative to the center of the base circle. This allows one to define a polarity of the drop via the in-plane unit vector (director) **d** which lies along the diametral stream and points towards the region of increased particle density (opposite to the orange arrow which illustrates the flow along that diameter, see Fig. 2c).

The flow pattern is maintained as long as the light source is on and sufficient fuel is in the drop. When turning the UV-light off, the flow stops within a few seconds and the system relaxes slowly towards the equilibrium state with a homogeneous monolayer of particles. Upon turning the UV-illumination on again, the flow pattern spontaneously re-forms, but the director **d** emerges in a different direction unrelated to the previous configuration (see Supplementary Movie 2). No stable state preserving the in-plane rotational symmetry has been observed, and repeated experiments lead to random orientations of the director. The phenomenology observed in a single drop is therefore neither the result of some external field acting on the CAPs or on the drop, nor a boundary effect. Instead, it is a genuine spontaneous breaking of the symmetry, leading to self-organized hydrodynamic flow in the drop and to collective motion of the particles.

Here we note that the range of areal number densities that have been explored and the duration of the experiment are limited by several factors. The catalytic chemical reaction at the surface of the particles consumes the peroxide and releases oxygen. At the same time, the concentration of peroxide must be kept low in order to avoid the formation of oxygen bubbles. Therefore, as the areal number density of particles is increased, the time which is available for the experimental observation of the system at steady state and in stable conditions (i.e., almost constant peroxide concentration) decreases, while the probability for the occurrence of oxygen bubbles significantly increases. As a consequence, the experiments reported here have been performed with area fractions of ≲40%, for which the dynamics is stable for at least 10 min. On the other hand, the transient time between the moment of turning on the UV-light and the establishment of the steady flow pattern increases with decreasing areal number density of CAPs, and the overall magnitude of the flow decreases while the particle accumulation along a diameter becomes less pronounced. For example, at an area fraction of ≈38% the steady pattern builds up within seconds after the UV-light is turned on, while at 12% area fraction this time increases to several minutes (see Supplementary Movie 3). At area fractions ≲4% we could not observe any sign of collective flow and motion. The apparent steep increase of the transient time is indicative of a sharp transition as a function of the area fraction in the spontaneous symmetry breaking.

Consequently, the question arises as to which mechanism leads to the self-organized collective motion of CAPs inside a drop. From experimental studies of bacteria^12–15^ or filament-molecular motor mixtures^2,5,16–19^ in films, drops, or microfluidic channels, as well as from theoretical and numerical studies devoted to such systems (see, e.g., refs. ^18,20–25^), it is now well established that, at sufficiently high densities, self-propelled particles with sufficiently large swimming velocities can form complex states of collective motion with polar order^21^ (active nematics also occurring; see,  e.g., the recent review in ref. ^26^). Such examples include swarms^2,5,12,15,18^, rotating vortices or bands^14,17^, and chaotic, active turbulence^27^. However, in our system only a fraction of the particles exhibit significant self-propulsion (see Fig. 1); moreover, those particles do not posses a defined polarity. Accordingly, self-propulsion cannot be the main factor contributing to the observed self-organized collective motion. Similarly, we can rule out pumping effects due to local density differences in the chemicals (see Supplementary Note 2). Rather, as discussed below, we find that the most plausible explanation is provided by the emergence of Marangoni flows owing to the local chemical gradients near the surface of the drop, which are induced by the chemical activity of the particles.

Remarkably, at a fluid interface, the chemical activity of a single microparticle is sufficient to induce gradients in the surface tension that cause significant Marangoni stresses and induce long-ranged Marangoni flows extending into the volume of the fluid. A single active particle of radius *R*_*p*_ located at a distance *L* *≳* *R*_*p*_ from an interface between two Newtonian fluids produces a Marangoni flow the magnitude of which can be estimated, following ref. ^28^, as (*Q b*_*0*_)/(64*πD*_+_ *η*_+_). Here *Q* denotes the total rate of product release by the particle, *η*_+_ denotes the average of the viscosities of the two fluids, *D*_+_ denotes the average of the diffusion constants in the two fluids of the product of the chemical reaction (i.e., O_2_ in the case of our particles), and *b*_0_ is the coefficient of tensioactivity relating, in linear approximation, the changes in the surface tension to the changes in the number density of the reaction product at the interface. By employing a reaction rate value comparable with the experimental report in ref. ^29^, assuming the diffusion constants of oxygen in water and in oil to be of the same order of magnitude (≈10^−9^ m^2^/s), and taking the coefficient *b*_0_ to have a value as small as 10^−3^ N/(m × M) (which is comparable to that of inorganic salts in water), one finds that a single micrometer–sized particle near a fluid interface is sufficient to induce a Marangoni flow at the interface with velocities of the order of tens of μm/s^28^. The effect is expected to be enhanced within a many-particle configuration, owing to the superposition of the flows induced by each of the particles^30^. This value favorably compares with current experimental observations: for drops in which ≈40% of the bottom surface is covered by CAPs, the particles near the contact line translate along the circumference with speeds of the order of 40 μm/s.

Moreover, the observed in-plane vortex structure is compatible with published reports of Marangoni flows in drops, e.g., in the experiments with a non-uniformly heated sessile drop^31,32^. The dynamic structure is also compatible with model calculations for fluid convection inside pairs of evaporating sessile drops located close to one another^33^, and with self-motile three-dimensional drops with micelle adsorption^34^ or with internally secreted surfactants^35^. As expected, we also observe flow in the oil phase near the outside of the drops (see Supplementary Note 1). The flow patterns (Supplementary Fig. 1c), as well as the measured tracer velocities (Supplementary Fig. 1d), are consistent with the observed flows near the interface within the drop. Although this does not provide additional insight into the underlying mechanism for the self-organized flows, it provides a good cross-check of the experimental observations.

Crucially, it is possible to unambiguously identify the emergence of activity-induced Marangoni flows as a necessary condition for the occurrence of the above phenomena via a complementary experiment: the water drop, containing peroxide and the particles, is left in contact with air instead of being immersed in oil. The very fast diffusion of oxygen in the surrounding air leads to practically a sink boundary condition for the oxygen at the surface of the drop and also raises *D*_+_ by orders of magnitude (up to ≈10^−4^ m^2^/s). This implies that the chemical gradient along the fluid interface is orders of magnitude smaller than in the case of a water-oil interface^28^. Therefore, one expects that in the case of the water-air interface the Marangoni flow is correspondingly reduced. When this complementary experiment is performed, one observes that, as shown in the Supplementary Movie 4, at the same large value of the area fraction and at the same concentration of peroxide, there is no indication, even after long times, for the emergence of a pattern of flow and a collective motion of the particles. However, the particles inside the drop exhibit motility upon UV illumination; therefore, the differences cannot be attributed to a lack of chemical activity, but they correlate with the predicted absence of the Marangoni flow response from the interface. Furthermore, it is known that for a water drop in air the evaporation of water may give rise to Marangoni stress and flow within the drop^36^. Thus the absence of the collective motion in this latter experiment implies that the activity-induced nature of these stresses is essential for the emergence of the phenomenology of the collective effects reported here. Accordingly, we conclude that the experiments reveal a novel mechanism of collective motion of active particles, which involves as main ingredients: the chemical activity of the particles, but not through self-motility, and the confinement of the system by a boundary which responds, via Marangoni stresses and flows, to the activity of the particles.

Furthermore, the theoretical predictions from ref. ^28^ also indicate that, if the drop is in contact with a phase with a very large viscosity compared to that of water, the Marangoni flow is very weak because the Marangoni stresses, localized at the surface of the drop, are suppressed. Upon increasing the viscosity of the outer oil phase from 5 to 10, 20, and 50 times that of water, we observe that the time for the polar patterns to occur systematically increases (see also the Supplementary Movie 5 and the Supplementary Fig. 3). Once the viscosity of the oil phase is increased to 100 times that of water, neither flow nor patterns of collective motion are observed. The predicted suppression of the Marangoni flows for large viscosities of the outer phase therefore correlates with the disappearance of the pattern of collective motion within the drop. This provides further support that the source driving the flow is at the fluid interface.

Once the crucial role played by the activity-induced Marangoni stresses on the interface has been elucidated, one may further speculate on the origin of the spontaneous symmetry breaking causing the observed phenomenology. A possible scenario is that of an instability in the transport of the chemical species at the water-oil interface, as discussed in ref. ^34^ in the context of drops in contact with micelles. (See also ref. ^31^ for experimental observations of a symmetry breaking of the Marangoni flow inside a locally heated water drop.) However, for our system this is not sufficient, because such an instability at the interface (i.e., one giving rise to a fore-aft asymmetry of the corresponding flow) would have to couple with a compatible in-plane clustering, driven by the in-plane component of that flow, of the active particles. An in-depth study of this complicated problem is deferred to future work.

### Cross-talk between many drops and emergence of ordered states

At this stage it is interesting to pose the question whether drops containing chemically active particles, which cause Marangoni flows, can also interact. Interactions between out-of-equilibrium drops have for instance been observed in arrays of bacteria-filled drops, in which the bacterial vortices inside neighboring drops give rise to hydrodynamic coupling^14^. Similarly, drops placed in air next to each other influence their evaporation rates, which results in relative motion due to Marangoni stresses^37,38^.

**Fig. 3:**
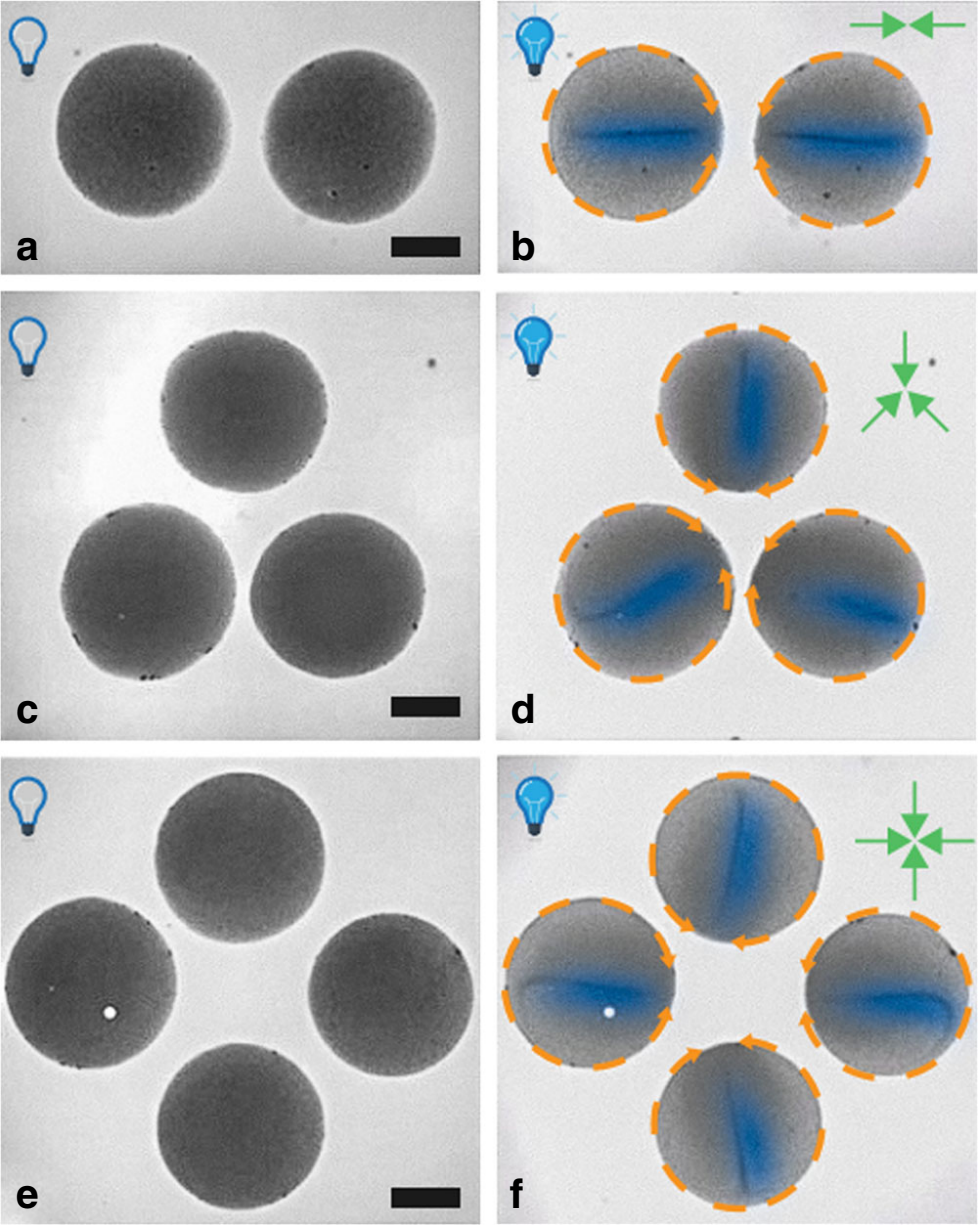
Alignment of the vortical flows in ensembles of active drops. Transition, upon illumination with UV light, from a uniform distribution of particles inside each drop (left column) to a steady hydrodynamic flow (schematically indicated by the dashed orange arrows), accompanied by the collective motion of the CAP within each drop (right column). The drops are arranged in regular, polygonal configurations. The panels on the right side illustrate the emergent alignment of the directors **d** of the drops: they point towards the geometrical center of the corresponding polygon, without any noticeable fluctuations in time. (For visual clarity, the region around a director is shaded in blue in accordance with the observed gray scale density.) The inset green arrows schematically depict the theoretically expected arrangement of the directors (see the main text). The scale bar corresponds to 100 μm.

In order to address this question, we place quasi-identical water drops containing CAPs in various geometrical arrangements, as shown in Fig. 3. Indeed, the drops can interact through the oil to influence the emerging flow patterns. While we do not observe any noticeable change in the dynamics inside a drop, clear deterministic global patterns emerge in the arrangements of the patterns inside the drops. The director of each drop always points approximately towards the geometrical center of the regular, polygonal arrangement (see Fig. 3 and Supplementary Movies 6, 7, and 8). Therefore, the active drops are able to sense each other and to respond by tuning their flow patterns.

In order to understand the underlying mechanism, we assume that the flow pattern and hence the orientation of the director **d** responds to the chemical and hydrodynamic fields of the other drops. Both the gradient in the distribution of chemicals and the strain in the flow create stresses at the surface of the drops, and each of these two can potentially bias the symmetry breaking without significantly altering the intra-drop dynamics. The latter observation indicates that these communication fields exert a small perturbation on a drop, which suggests that a far-field approximation can be used in order to estimate the chemical and hydrodynamic fields (see Supplementary Note 3 for details). Accordingly, each drop is approximated as a monopolar source for chemicals and a monopolar source for the flow in the oil (i.e., a Stokeslet), located very close to the bottom wall. The Stokeslet is due to the fact that the active drop, within which the steady flow has been established, is unable to move because its contact line is pinned. The presence of the wall imposes boundary conditions of zero flux for the chemicals and no slip for the flow. The latter strongly diminishes the role hydrodynamics plays in the coupling, and suggests that the dominant mechanism for the drop–drop communication is mediated via the chemical field. In view of the mathematical analogy between the Poisson equation describing the distribution of the number densities of chemical species and Newtonian gravity, one can think of each drop effectively possessing a mass and the chemical gradients to be the gravitational field. Consequently, for any drop the director **d** is predicted to exhibit the direction of the gravitational field created by the masses at the center of each drop. Whether **d** is parallel or anti-parallel to the field is deduced by comparison with a single experiment (see Supplementary Note 3); we find that the director **d** aligns parallel to the gradient of the chemical field.

In order to test experimentally the role of an external influence, we have placed an active drop next to a nearby inactive drop containing water and titania particles, but no hydrogen peroxide. Neither motion of the particles, nor the emergence of collective flows are observed within the inert drop, whereas the pattern formation in the active drop is basically insensitive to the presence of the inert drop (see Supplementary Movie 9). Accordingly, the absence of particle motion emphasizes the need for internal chemical activity for the emergence of the collective motion and inter-drop communication.

**Fig. 4:**
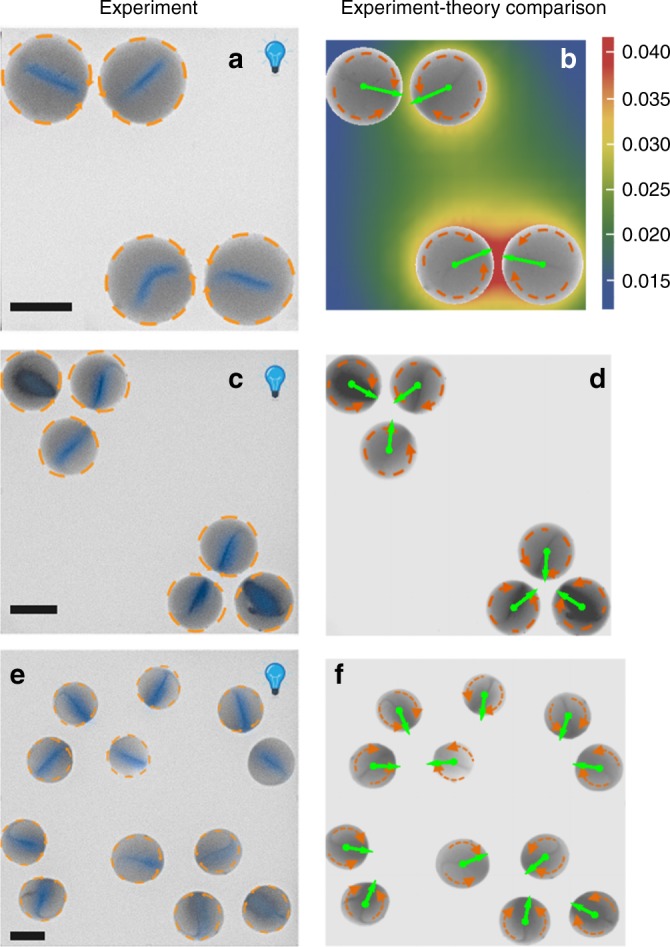
Experimental observations and theoretical prediction of the flow directors. The emergent alignment of the directors of the drops, forming various configurations with low symmetry, caused by the superposition of long-ranged pair communication (see Supplementary Note 3). Left column: experiment (for visual clarity, the region around the director is shaded in blue in accordance with the observed gray scale density); right column: comparison with the theoretical prediction (green arrows, see Supplementary Note 3) with the activity strength *Q*_i_ taken to be proportional to the area of the base of the *i*-th drop. The scale bars in the left panels correspond to 150 μm. The color background in the top right panel depicts the chemical field (here O_2_), in units of *Q*/*R*, that determines the orientation of the director of the top left drop, i.e., the number density of the chemical due to the other three drops in that panel.

In view of the complexity of the behavior within the drops, it is somewhat surprising that this simple theoretical description of the system quasi-quantitatively captures the observed ordering of the directors **d** in ensembles of drops. The model clearly reproduces the behavior exhibited by quasi-identical drops in Fig. 3. A stronger test of the predictive power of the model is provided by ensembles of quasi-identical drops either in configurations with low symmetry, or randomly distributed in the lateral directions. Experimental results, together with the corresponding theoretical prediction for the ordering of the directors (see Supplementary Note 3), are shown in Fig. 4 and via the Supplementary Movies 10, 11, and 12. There is qualitatively good agreement between the predictions of the model and the experimental observations. Especially the case in which the drops are randomly distributed provides particularly strong support for the simple theoretical model; deviations thereof could arguably be attributed to corrections to the far-field approximation, hydrodynamic corrections, as well as imperfections in the experiment.

**Fig. 5:**
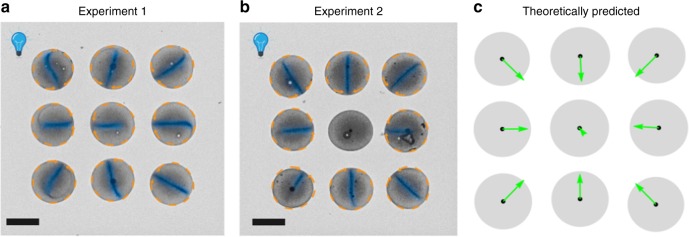
Alignment of the flow directors for arrays with high symmetry. The emergent alignment of the directors of drops arranged in square arrays of size 3 × 3 for two independent experiments (left and middle panel; for visual clarity, the region around the director is shaded in blue in accordance with the observed gray scale density). The right panel shows the theoretically predicted chemical gradients, i.e., the director **d** before normalization (see Supplementary Note 3) with the activity strength *Q*_i_ taken to be proportional to the area of the base of the *i*-th drop. The differences in the emergent orientations of the directors between the two repeats of the experiment are clearly noticeable in the central part of the ensemble. The scale bars correspond to 150 μm.

Another testable prediction of the theoretical model concerns the issue of how the director behaves at positions where the calculated chemical gradient is almost vanishing. This is the case in some highly symmetric configurations of many drops and can be seen in Fig. 5 for ordered 3 × 3 arrays of drops (see Supplementary Fig. 4 for similar results concerning 4 × 4 arrays of drops). Experimentally, one indeed observes, particularly at the places where **d** is predicted to vanish, both a lack of a deterministic outcome concerning the orientation of **d**, as well as a sensitivity of the emerging alignment to minor perturbations (see, e.g., the central drop in Fig. 5). The theoretical model, as before, captures quite well the ordering of the directors for the drops at the fringes (see the right columns in the panels in Fig. 5, and note that here the lengths of the arrows show the relative magnitude of the gradients). At positions where the predicted gradient almost vanishes, the experimental results suggest that the interdrop communication, falls below the threshold of the omnipresent uncontrollable perturbations so that the orientation of **d** becomes random, as in the experiments for an isolated drop.

Thus, our experiments and the theoretical analysis demonstrate that drops containing chemically active particles show chemical communication with respect to the ordering of their internal patterns of flow and collective motion, which is surprisingly long-ranged and robust.

## Discussion

Chemical activity of particles sedimented at the bottom of a sessile drop resting on a support produces changes in the chemical composition of the drop. These can alter the surface tension of the drop in a spatially inhomogeneous manner. Even relatively small gradients in chemical composition can induce relevant Marangoni stresses at the surface of the drop. In turn, these can give rise to significant flows that extend into the volume of the drop and couple back to the particles. Even though the self-propulsion of the particles is small, the Marangoni flows induced by their activity give rise, within the drop, to a global symmetry breaking and to the emergence of self-organized, polar patterns of flow as well as to a complex dynamics of the sedimented particles.

When a single, isolated drop is considered, we observe that upon sufficiently increasing the areal particle density the homogeneous particle distribution is spontaneously replaced by an inhomogeneous quasi-steady particle distribution characterized by a well-defined in-plane orientation (expressed in terms of a director **d**). This is accompanied by a similarly well-defined pattern of hydrodynamic flow exhibiting two symmetric vortices (see Fig. 2). The direction of the director is a random variable with respect to repeats of the experiment, suggestive of a spontaneous symmetry breaking. This phenomenology thus reveals a new mechanism of collective motion, which involves triggering a hydrodynamic response of a confining interface through the chemical activity of the particles. The bidirectional coupling between the Marangoni flow that advects the particles, which themselves create the Marangoni stresses through the release of chemicals, poses a difficult mathematical problem that has prevented us from a complete analysis of the feedback mechanism.

If an ensemble of neighboring active drops is considered, we observe that the director **d** of each drop has no longer a random, unpredictable orientation. Instead, the orientation is a robust feature and can be fully rationalized by assuming that the stresses on the fluid surface of any one drop are biased by the neighboring drops. This external stress is due to the gradients in the chemical composition and to the strain in the flow occurring in the medium between the drops. Within the far-field approximation, which is consistent with the observation that this effect is a small perturbation (because the dynamics inside each drop seems to be qualitatively unaltered), we could show that the dominant contribution is the gradient in the chemical composition, rather than the hydrodynamic contribution. Accordingly, a collection of several such active drops can exhibit mutual chemical communication mediated by the surface stress response of the drops. Our model captures quasi-quantitatively the emerging deterministic orientations of the directors in various ensembles of drops.

The intriguing question arises as to whether such particle-loaded drops would steadily self-propel if they were not pinned. When the drop is pinned, we have observed symmetry breaking and the formation of a polar pattern. If such a drop, with the polar pattern already formed, would be suddenly unpinned, we expect that it would start to move. However, it is not clear if such a motion would persist (self-motility) or whether the drop would evolve to a non-motile state. Elucidating the role played by the pinning of the drop (i.e., a fixed confining interface) in the course of the emergence of symmetry breaking is both experimentally and theoretically very challenging; we, therefore, leave this question for future studies.

In summary, our study illustrates how simple active particles that show little self-motility can give rise—if present in large numbers and at high number density—to macroscopically ordered systems, i.e., active drops, which can then interact and thereby self-organize. A simple application of such active drops would be to use them as on-demand micro-mixers or micro-pump devices for the outer solution: when the drop is illuminated, the inside flows set in and — owing to the coupling to the outer fluid via the interface—drive flow in the outer fluid (here the oil phase). By using well-designed arrangements of such small drops, and their individual addressing by illumination, complex topologies of the flow in the outer phase could be created.

## Methods

### Chemically active powders and drops

We use commercial photo-chemically active anatase TiO_2_ particles (US Research Nanomaterial Inc.) with a diameter of approximately 1 μm on average. The particles have somewhat anisotropic shapes, as can be inferred from the optical microscopy image shown in Supplementary Fig. 1a. Their surface is uniform in terms of chemical activity, i.e., the UV photo-catalytic decomposition of hydrogen peroxide from an aqueous solution. In the absence of UV illumination, the particles are chemically inert.

In the experiment, a well mixed suspension is prepared by dispersing 2 mg of TiO_2_ particles in 10 mL of aqueous peroxide solution (volumetric concentration 3% v/v). Subsequently, a funnel-shaped glass capillary tube is filled with the solution and is then used to cast sessile drops on a glass cover slip by contacting the glass. Using a capillary tube which accommodates a sufficiently large volume (≈10 μL) of the suspension, a large number of quasi-identical drops, each of ≈0.01 μL (diameter ≈ 300*μ*m), can be formed on the glass slide. As long as the volume of the solution inside the capillary is much larger than that of the dispensed drop, the volume of the drop depends only on the opening diameter of the capillary. This is the case of our experiments, in which up to 25 such drops have been used. The positioning of the drops is precisely controlled using an X–Y stage manipulator (Physik Instrumente GmbH & Co. KG). Once the drops are formed on the glass slide, the sample is sealed with a walled plastic chamber of width and length much larger than the region occupied by the drops (the walls of the chamber are at least 10 diameters away from the center of the nearest drop). Finally, the drops are covered with silicon oil of kinematic viscosity 5 cSt (Sigma Aldrich) by gently pouring the oil into the chamber.

### Illumination and video recording

The dense TiO_2_ particles sediment to the bottom glass slide. Once sedimented, one observes, as expected, a uniform areal distribution of particles at the bottom glass plate (see Supplementary Fig. 1a). This is the initial state for all studies of the motion of the particles. In order to turn on the photo-catalytic activity of the particle, which drives the system out of equilibrium, the system is exposed to UV light (wavelength 365 nm) from an emitting diode in an epifluorescence-type arrangement. Under these conditions, TiO_2_ acts as a catalyst for the decomposition of peroxide into water and oxygen (see, e.g., refs. ^7–10^). The motion of the particles is recorded at 20 frames/s for subsequent analysis by using an inverted optical microscope in transmittance mode.

### Intensity and concentration dependence

Increasing the peroxide concentration can also increase the activity and motility of the particles. However, for a photo-catalytic system, the activity is limited by the available light intensity (which determines the creation of electron-hole pairs) as well as by the concentration of fuel (here H_2_O_2_). For a fixed light intensity, with the largest possible value of 439 mW/cm^2^, the motility of the particles increases with the concentration of H_2_O_2_ up to 3% v/v H_2_O_2_, beyond which it levels off (see Supplementary Fig. 1b and the Supplementary Movie 13).

## Supplementary information

Supplementary Information

Description of Additional Supplementary Files

Supplementary Movie 1

Supplementary Movie 2

Supplementary Movie 3

Supplementary Movie 4

Supplementary Movie 5

Supplementary Movie 6

Supplementary Movie 7

Supplementary Movie 8

Supplementary Movie 9

Supplementary Movie 10

Supplementary Movie 11

Supplementary Movie 12

Supplementary Movie 13

Supplementary Movie 14

## Data Availability

The data that support the findings of this study are available from the corresponding authors upon reasonable request.
