# Supplementary material for: Interface-mediated spontaneous symmetry breaking and mutual communication between drops containing chemically active particles
Source: Nat Commun. 2020 May 5;11:2210. doi: 10.1038/s41467-020-15713-y (PMC7200706; doi:10.1038/s41467-020-15713-y)
Supplement: Supplementary file 1 — Supplementary Information [file 41467_2020_15713_MOESM1_ESM.pdf]

Interface-mediated spontaneous symmetry breaking and mutual communication between drops containing chemically active particles

Supplementary Information

D.P. Singh,[1,2,*] A. Domínguez,[3,†] U. Choudhury,[1]
S.N. Kottapalli,[1] M.N. Popescu,[1] S. Dietrich,[1,4] and P. Fischer[1,5,‡]

[1] *Max-Planck-Institut für Intelligente Systeme, Heisenbergstr. 3, D-70569 Stuttgart, Germany*
[2] *Department of Physics, Indian Institute of Technology Bhilai, Raipur-492015, India*
[3] *Física Teórica, Universidad de Sevilla, Apdo. 1065, 41080 Sevilla, Spain*
[4] *IV. Institut für Theoretische Physik, Universität Stuttgart, Pfaffenwaldring 57, D-70569 Stuttgart, Germany*
[5] *Institut für Physikalische Chemie, Universität Stuttgart, Pfaffenwaldring 55, D-70569 Stuttgart, Germany*

---

* dhruv@iitbhilai.ac.in
† dominguez@us.es
‡ fischer@is.mpg.de

Supplementary Note 1: Additional experimental data

Flow around the active drop

As discussed in the main text, at large areal densities of titania particles patterns of collective flow and motion occur within the drop. These are accompanied by flow within the outer oil phase, as shown by the motion of polylactic acid (PLA) tracer particles near an active drop (see the Supplementary Figure 1 (c) and the Supplementary Movie 14). Furthermore, from the Supplementary Movie 14 we have extracted 20 trajectories, and we have determined the velocities of the tracers along the streamlines surrounding the drop in two regions near the stagnation point. These data are shown in Supplementary Figure 1 (d) as a function of distance from the interface of the drop; it confirms that the maximal velocities (~ 26 μm/s) are, as expected, somewhat smaller than the observed flows near the interface within the drop.

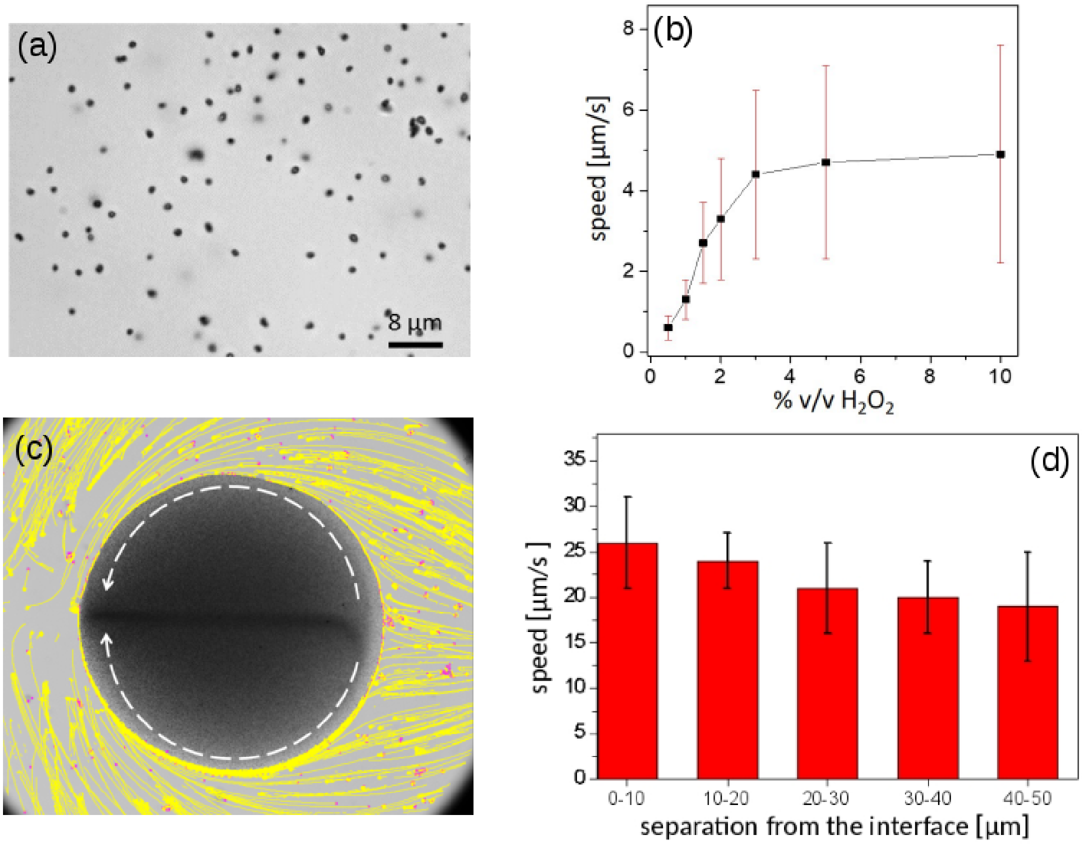

Supplementary Figure 1. (a) Optical microscopy image of anatase TiO_2 particles sedimented at a glass substrate. The blurry spots are particles which are not sedimented, and thus their images are out of focus. (b) Motility of individual TiO_2 particles as a function of the H_2O_2 concentration. Each data point is an average over 30 particles. The error bars are given by the standard deviation. (c) Streamlines in the oil around the active drop, illustrated by the trajectories of PLA tracers (2 μm diameter). The red spots show tracer particles in the oil phase. (Note that only some of the tracer particles are motile, while others are stuck to the glass slide.) (d) Speed of tracer particles as a function of distance from the interface. Each data point is an average over five tracers. The error bars are given by the standard deviation.

Image analysis

In the cases in which a pattern of collective motion occurs, image analysis is performed as described below in order to determine the time for the emergence of the pattern inside an active drop (see also Supplementary Figure 2). In each case, the frames of the video are rotated such that the director is aligned along the vertical (y-axis). A rectangular region at the center is selected and the images are cropped. The first 100 frames (well before the UV light is turned on) are used to determine the background intensity (i.e., the average of the gray values of the pixels), which is subtracted from all the subsequent frames. Then, in each frame the average intensity along the x-axis is determined in order to obtain a line of average intensity for which the standard deviation σ is determined. The plot of the time-dependent

evolution of the standard deviation $\sigma(t)$ is shown in Supplementary Figure 2. The emergence of a pattern inside a drop is signaled by the increase in the standard deviation as a result of the systematic change of the fluctuations in the pixel intensity inside the rectangular region. The time of occurrence is then set by the intersection of the line fitting the horizontal plateau at early times with a linear fit to the increasing $\sigma(t)$ (see Supplementary Figure 2).

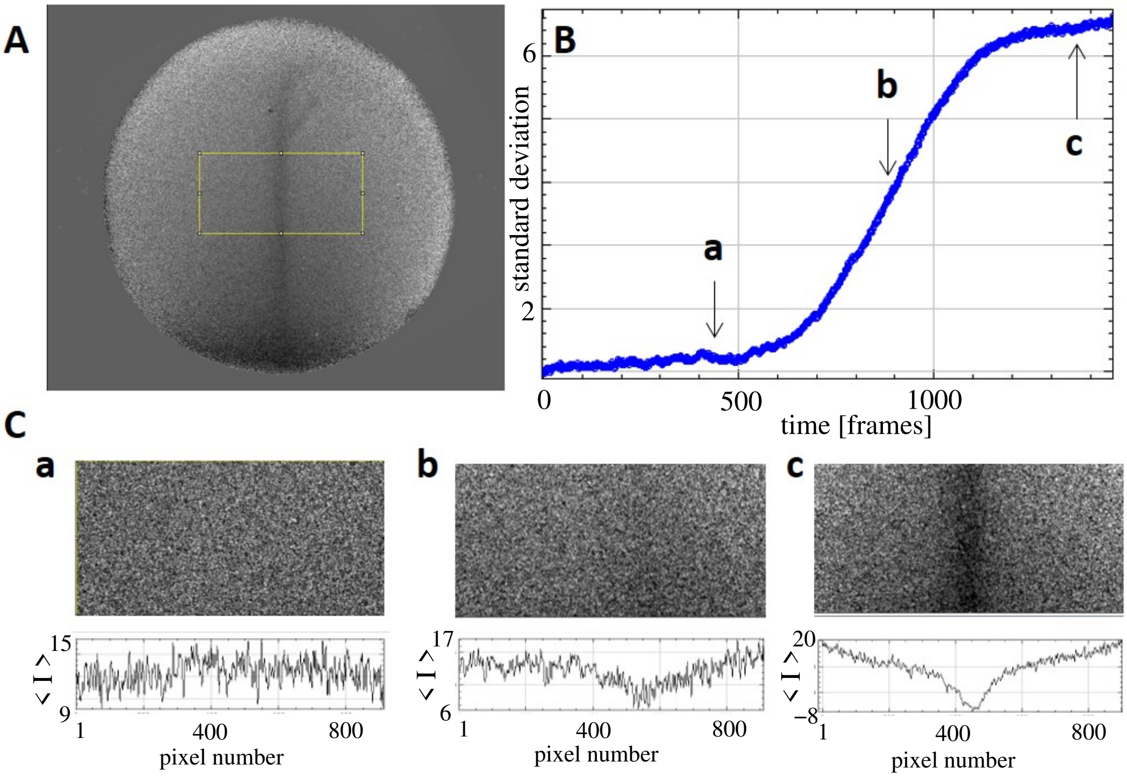

Supplementary Figure 2. Illustration of the image analysis. A) Selection of the central area of the drop after rotation. B) Plot of the time-dependent evolution of the standard deviation. C) For the points a, b, c indicated in B), respectively, the corresponding images (i.e., at the corresponding times) of the selected area are shown. Underneath, the plots of the corresponding line intensity (i.e., the average across the image, see the text for details) are given.

This procedure has been applied to determine the time for the emergence of the pattern as a function of the peroxide concentration (Supplementary Figure 3(a)) and as a function of the viscosity of the outer oil phase (Supplementary Figure 3(b)).

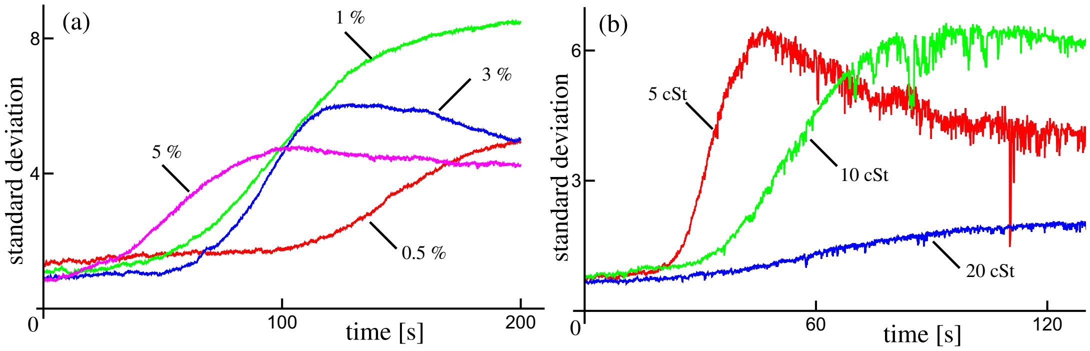

Supplementary Figure 3. (a) Analysis of the emergence of the director as a function of the H_2O_2 concentration for drops. It is seen that the pattern generally emerges faster for higher H_2O_2 concentrations. The computation of the standard deviation is based on a frame-by-frame analysis of the corresponding Supplementary Movie 4. (b) Emergence of the patterns as a function of the viscosity of the surrounding oil phase. The time it takes for the patterns of collective motion to emerge increases with increasing oil viscosity, and the onset times are approximately 20s for 5 cSt, 30s for 10 cSt, and a much slower response for 20 cSt.

Supplementary Note 2: Further consideration of mechanisms underlying collective motion of CAPs inside a drop

Irrespective of whether or not there is a genuine dynamic transition occurring at a certain threshold value of the area fraction, there are several factors involved in the emergence of this self-organized non-equilibrium steady state. Clearly, the area fraction, the geometrical confinement, and the chemical activity of the particles are relevant factors. The latter can potentially contribute — in addition to the mechanism of inducing Marangoni flows as discussed in the main text — in several other ways, including the chemophoresis of the particles or buoyancy-induced flows due to changes in the chemical compositions, which are discussed below.

A particle can exhibit chemophoresis due to chemical gradients induced by its neighbors. (This is the same mechanism as the one applicable for self–propulsion, in which case the gradients are induced by the deviation from the spherical shape of the particle itself.) However, owing to the decay of the chemical gradient as function of the distance from the source, the single-particle effect is expected to be at most of the order of the self-propulsion velocity which the particle would have exhibited if it was not spherical($\approx$ 5 μm/s according to our measurements quoted above). In addition, its collective influence due to many-particle superposition is expected to be weaker than in the case of Marangoni flow. This is because the Marangoni velocity decays like $1/r$ as function of the separation from its source (as inferred from Green's function of the Stokes equation for the flow), whereas the chemophoretic velocity decays like $1/r^2$ (as inferred from the gradient of Green's function of the Laplace equation for the distribution of chemicals).

It has been shown in the context of "active" pumps (see, e.g., Refs. [1–3]) that the presence of spatially extended patches of chemical activity can give rise to convective flows in the liquid cell. This can be either due to osmotic flows along the wall on which the active patch is imprinted (as it was argued for electro-catalytic pumps based on peroxide decomposition [1, 3]), or due to buoyancy-driven flows caused by the formation of a lighter and faster diffusing product species from a heavier and slower diffusing reactant species (as it was argued for enzymatic pumps [2]). Such phenomena could be relevant if one would argue that a cluster of particles acts as a chemically active patch pumping the fluid. However, the observed topology of the in-plane flow (two vortices, polar pattern) is significantly different from that of an in-plane radial flow, from the contact line region towards a center located at the regions of high densities of particles (or opposite), as it would be expected for convective flows as the ones discussed above. Accordingly, we infer that this mechanism cannot be the main cause of the phenomenology exhibited by our system.

Supplementary Note 3: Theoretical model of interdrop cross–talk

In addition to the Marangoni stresses experienced by a drop due to the activity of the particles it contains, as studied here, each drop is also exposed to stresses at its surface due to the gradients in composition and the flow strains in the oil caused by neighboring "active drops". First we consider the distribution of chemicals (chemical field) . It is assumed to be given by the solution of the Poisson equation, expressing "diffusive equilibrium", with the sources located inside the drops and with the boundary condition of no flux of chemicals through the bottom, solid wall. In the far-field approximation, each drop is approximated as a monopole of strength $\mathcal{Q}$ (i.e., the total rate of chemical production by all the particles contained in a single drop) positioned at the center of mass of the drop, which is located at a small height h above the bottom wall. (For the typical height $\simeq 100$ μm of the drop of radius $\simeq 150$ μm, the height h of the center is roughly ≈ 36 μm [4].) Although $\mathcal{Q}$ can be positive or negative (depending on whether the particles are sources or sinks of the relevant chemicals), we simply take it to be positive (i.e., we consider $|\mathcal{Q}|$), because the eventual dynamical effect of the chemical gradient depends in addition on other factors which we ignore, e.g., whether the chemicals have a negative or positive tensioactive effect (i.e., the surface tension increases or decreases with increasing concentration of chemicals at the interface), and how the gradients eliminate the orientational degeneration of the director. As stated in the main text, the ambiguity related to the orientation of the director being parallel or antiparallel to the chemical gradient produced by the other drops is removed by studying a specific, single experiment.

Therefore, the perturbation of the number density of the chemical produced by the activity of the i-th drop (modeled as a monopole of strength $\mathcal{Q}_i$ located at $\mathbf{R}_i$) is proportional to $|\mathcal{Q}|/|\mathbf{r}-\mathbf{R}_i|$ in the limit $h \ll |\mathbf{r}-\mathbf{R}_i|$, where $\mathbf{r}$ is the position of the "observation point". The gradient of this field induces gradients in the surface tension of the fluid interface of the other drops, which produces the Marangoni stresses that ultimately determine the orientation of the director. Therefore, the influence of the chemical field, within this far-field approximation, on the director $\mathbf{d}_k$ of the k-th drop leads to the result

$$\mathbf{d}_k \text{ is oriented along } \sum_{i\neq k}\left(\nabla_{\mathbf{r}}\frac{|\mathcal{Q}_i|}{|\mathbf{r}-\mathbf{R}_i|}\right)_{\mathbf{r}=\mathbf{R}_k}. \tag{1}$$

By considering the outcome of a specific, single experiment involving multiple drops, such as the two-drop configuration in Fig. 3 (main text), it follows that $\mathbf{d}_k$ is parallel (rather than antiparallel) to this superposition of gradients. Thus, concerning the emerging orientations of the directors, one can consider each drop as a "mass" of magnitude $|\mathcal{Q}|$, and the gradient of the chemical field as the "gravitational field" created by these "masses".

We now consider the effect of the flow in the oil. This flow is given by the solution of the Stokes equation, which is expressing "force equilibrium", together with the no-slip boundary condition at the bottom wall and with the drops acting as sources of momentum. The directional character of the flow pattern inside a drop in our experiments does actually suggest that the drop would self-propel, i.e., slide along the wall if free (this indeed is the case for drops in bulk fluid, see, e.g., Refs. [5, 6]). This does not occur for the sessile drops studied here because they are pinned by the bottom wall at the contact line. This implies that the wall exerts a net force $\mathbf{F}_{\text{pin}}$, in the lateral direction, on the liquid. Thus the *immobilized* drop is modeled within the far-field approximation as an in-plane Stokeslet of strength $\mathbf{F}_{\text{pin}}$ (i.e., a hydrodynamic monopole), also located at the center of the drop. Expressed in cylindrical coordinates such that the Stokeslet is located at $h\mathbf{e}_z$ and the wall is the plane $z = 0$, the velocity field induced by this Stokeslet at the position $\mathbf{r} = \rho\mathbf{e}_\rho + z\mathbf{e}_z$ is proportional to the expression

$$\frac{4h}{\rho^2}\left[\frac{3z}{\rho}\mathbf{e}_\rho - \mathbf{e}_z\right](\mathbf{e}_\rho \cdot \mathbf{F}_{\text{pin}}), \qquad (2)$$

to lowest order in the small quantity $1/\rho$ [7–9]. Therefore, the in-plane component of both the flow and the strain at the position $\mathbf{R}_k$ of one drop due to a second drop located at $\mathbf{R}_i$ scales as $F_{\text{pin}}h/|\mathbf{R}_k - \mathbf{R}_i|^3$. Within the far-field approximation ($h \ll |\mathbf{R}_k - \mathbf{R}_i|$), this contribution can be expected to be much smaller than the one due to the chemical field. Therefore, in this simplified theoretical model one can neglect this contribution. Accordingly, the orientation of the director of the drops is determined solely by the effective communication due to the chemical field (see Supplementary Equation (1)).

Supplementary Note 4: Arrangement of directors in ordered 4×4 arrays of drops

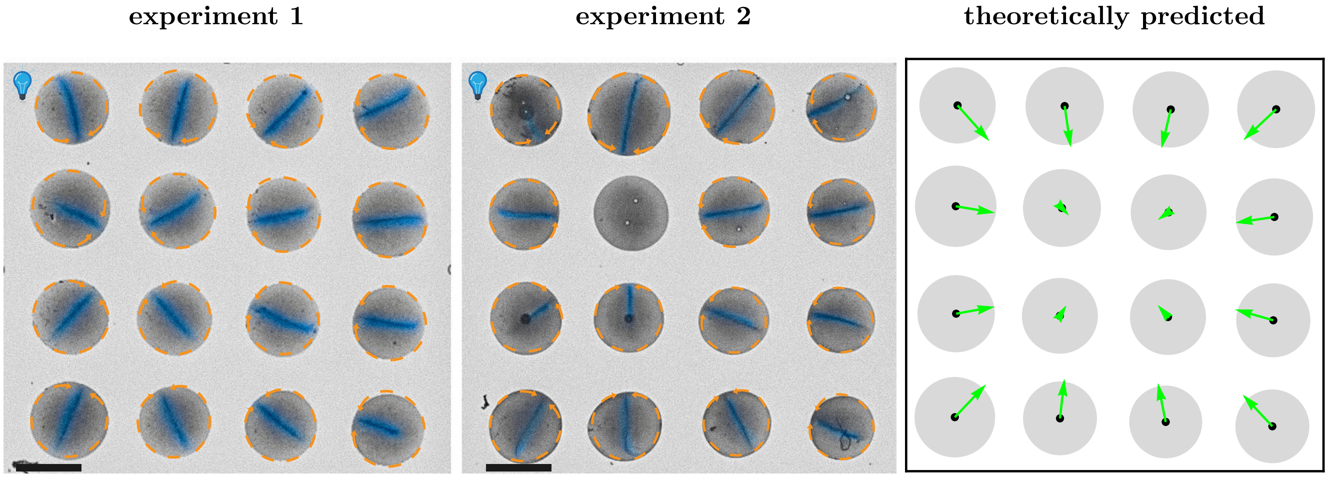

Supplementary Figure 4. The emergent alignment of the directors of drops arranged in square arrays of size 4×4 for two independent experiments (left and middle panels; for visual clarity, the region around the director is shaded in blue in accordance with the observed gray scale density). The right panel shows the theoretically predicted chemical gradients, i.e., the director $\mathbf{d}$ before normalization (Supplementary Equation (1) with the activity strength $\mathcal{Q}_i$ taken to be proportional to the area of the base of the i-th drop). The differences in the emergent orientations of the directors between the two repeats of the experiment are clearly noticeable in the central part of the ensemble (compare the left and the middle panels). The scale bars correspond to 150 μm.

Supplementary References

---

[1] Kline, T. R. *et al.* Catalytic micropumps: microscopic convective fluid flow and pattern formation. *J. Am. Chem. Soc.* **127**, 17150–17151 (2005).

[2] Ortiz-Rivera, I., Shum, H., Agrawal, A., Sen, A. & Balazs, A. C. Convective flow reversal in self-powered enzyme micropumps. *Proc. Nat. Acad. Sci.* **113**, 2585–2590 (2016).

[3] Farniya, A. A., Esplandiu, M. J., Reguera, D. & Bachtold, A. Imaging the proton concentration and mapping the spatial distribution of the electric field of catalytic micropumps. *Phys. Rev. Lett.* **111**, 168301 (2013).

[4] Harris, J. W. & Stocker, H. *Handbook of Mathematics and Computational Science* (Springer, New York City, 1998).

[5] Thutupalli, S., Seemann, R. & Herminghaus, S. Swarming behavior of simple model squirmers. *New J. Phys.* **13**, 073021 (2011).

[6] Schmitt, M. & Stark, H. Marangoni flow at droplet interfaces: three-dimensional solution and applications. *Phys. Fluids* **28**, 012106 (2016).

[7] Blake, J. R. & Chwang, A. T. Fundamental singularities of viscous flow. *J. Eng. Math.* **8**, 23–29 (1974).

[8] Aderogba, K. & Blake, J. Action of a force near the planar surface between two semi-infinite immiscible liquids at very low Reynolds numbers. *Bull. Austral. Math. Soc.* **18**, 345–356 (1978).

[9] Jones, R. B., Felderhof, B. U. & Deutch, J. M. Diffusion of polymers along a fluid-fluid interface. *Macromolecules* **8**, 680–684 (1975).
