# Supplementary material for: Interface-mediated spontaneous symmetry breaking and mutual communication between drops containing chemically active particles
Source: Nat Commun. 2020 May 5;11:2210. doi: 10.1038/s41467-020-15713-y (PMC7200706; doi:10.1038/s41467-020-15713-y)
Supplement: Supplementary file 2 — Description of Additional Supplementary Files [file 41467_2020_15713_MOESM2_ESM.pdf]

Description of Additional Supplementary Files

1. Supplementary Movie 1: Spontaneous symmetry breaking and emergence of vortical flows inside a single drop. The areal number density of particles is $\approx$ 40% and the drop consists of an aqueous solution of 3% (v/v) hydrogen peroxide (H_2O_2). The viscosity of the oil is 5 cSt. The video is sped up by a factor of 3.

2. Supplementary Movie 2: Random orientation of the vortical flows and the director in a single drop. The areal number density of particles is $\approx$ 40% and the drop consists of an aqueous solution of 3% (v/v) hydrogen peroxide (H_2O_2). The viscosity of the oil is 5 cSt. The video is sped up by a factor of 4.

3. Supplementary Movie 3: Active drops of various areal number density of particles (4%, 12%, and 38%). The drops consist of an aqueous solution of 3% (v/v) hydrogen peroxide (H_2O_2). The viscosity of the oil is 5 cSt. The video is sped up by a factor of 10.

4. Supplementary Movie 4: Active drop without immersion in oil medium (exposed to air). The areal number density of particles is $\approx$ 40% and the drop consists of an aqueous solution of 3% (v/v) hydrogen peroxide (H_2O_2). The video is sped up by a factor of 2.

5. Supplementary Movie 5: Active drops in contact with oils of different viscosities (5 cSt, 20 cSt, and 50 cSt). The areal number density of particles is $\approx$ 40% and the drops consist of an aqueous solution of 3% (v/v) hydrogen peroxide (H_2O_2). The video is sped up by a factor of 2.

6. Supplementary Movie 6: Two active drops. The areal number density of particles is $\approx$ 40% and the drops consist of an aqueous solution of 3% (v/v) hydrogen peroxide (H_2O_2). The viscosity of the oil is 5 cSt. The video is sped up by a factor of 5.

7. Supplementary Movie 7: Three active drops arranged in a triangular pattern. The areal number density of particles is $\approx$ 40% and the drops consist of an aqueous solution of 3% (v/v) hydrogen peroxide (H_2O_2). The viscosity of the oil is 5 cSt. The video is sped up by a factor of 5.

8. Supplementary Movie 8: Four active drops arranged in a square pattern. The areal number density of particles is $\approx$ 40% and the drops consist of an aqueous solution of 3% (v/v) hydrogen peroxide (H_2O_2). The viscosity of the oil is 5 cSt. The video is sped up by a factor of 5.

9. Supplementary Movie 9: An active drop placed next to an inactive drop containing water and titania particles, but no hydrogen peroxide. The areal number density of particles is $\approx$ 40% and the active drop consists of an aqueous solution of 3% (v/v) hydrogen peroxide (H_2O_2). The viscosity of the oil is 5 cSt. The video is sped up by a factor of 2.

10. Supplementary Movie 10: Four active drops arranged as two pairs (doublets at a distance). The areal number density of particles is $\approx$ 40% and the drops consist of an aqueous solution of 3% (v/v) hydrogen peroxide (H_2O_2). The viscosity of the oil is 5 cSt. The video is sped up by a factor of 5.

11. Supplementary Movie 11: Six drops arranged as a pair of triangles (two triangles at a distance). The areal number density of particles is $\approx$ 40% and the drops consist of an aqueous solution of 3% (v/v) hydrogen peroxide (H_2O_2). The viscosity of the oil is 5 cSt. The video is sped up by a factor of 4.

12. Supplementary Movie 12: Randomly placed active drops. The areal number density of particles is $\approx$ 40% and the drops consist of an aqueous solution of 3% (v/v) hydrogen peroxide (H_2O_2). The viscosity of the oil is 5 cSt. The video is sped up by a factor of 4.

13. Supplementary Movie 13: Active drops with different peroxide concentrations (1%, 3%, and 5% (v/v)). The areal number density of particles is $\approx$ 40%. The viscosity of the oil is 5 cSt. The video is sped up by a factor of 2.

14. Supplementary Movie 14: Motion of tracer particles (2 μm diameter PLA (polylactic acid)) around an active drop, within the oil phase. The areal number density of titania particles within the drop is $\approx$ 40% and the drop consists of an aqueous solution of 3% (v/v) hydrogen peroxide (H_2O_2). The viscosity of the oil is 5 cSt.
